# Scientific approach to derive occupational exposure limits for formaldehyde releasers

**DOI:** 10.1007/s00204-026-04489-7

**Published:** 2026-07-03

**Authors:** Sandra Michaelsen, Britta Laube, Rüdiger Bartsch, Stephan Baumgärtel, Dietmar Breuer, Rolf Grebenstein, Edgar Leibold, Ruth Lohmann, Hans Merk, Carsten Schleh, Manigé Fartasch, Michael Bader, Hans Drexler, Brunhilde Blömeke, Andrea Hartwig

**Affiliations:** 1 Department of Food Chemistry and Toxicology, Institute of Applied Biosciences (IAB), Karlsruhe Institute of Technology (KIT), Karlsruhe, Germany; 2 Verband Schmierstoff-Industrie e.V, Hamburg, Germany; 3 Hennef, Germany; 4 Immenstadt, Germany; 5 Carlsberg, Germany; 6 Department of Dermatology and Allergology, Medical Faculty, RWTH Aachen University, Aachen, Germany; 7 Abteilung Gesundheit, Berufsgenossenschaft Holz und Metall, Munich, Germany; 8 Dermatology Private Practice Siegburg, Siegburg, Germany; 9 BASF SE, Ludwigshafen, Germany; 10 Institute and Outpatient Clinic of Occupational, Social and Environmental Medicine, Friedrich-Alexander-Universität Erlangen-Nürnberg (FAU), Erlangen, Germany; 11 Department of Environmental Toxicology, Trier University, Trier, Germany

**Keywords:** Formaldehyde releasers, Carcinogenicity, Irritation, Skin absorption, Sensitization, Occupational exposure limit

## Abstract

Formaldehyde is highly reactive, causing irritation and acute and chronic toxicity in target tissues (the eyes, respiratory tract, and skin) following inhalation and/or skin contact. Moreover, formaldehyde is a local carcinogen after inhalation exposure. It induces nasal squamous cell carcinoma in laboratory animals and nasopharyngeal carcinoma in humans, albeit less convincingly. While formaldehyde has been evaluated and classified by different organizations and committees in recent years, the question has arisen of how to deal with substances that release formaldehyde. These releasers decompose in aqueous media and are frequently used as biocides in water-miscible metalworking fluids, adhesives, paints, disinfectants, and cosmetics. In this context, the MAK Commission has developed a concept for differential evaluation regarding carcinogen classification and MAK value derivation. This approach is based on hydrolysis rates as a function of pH, temperature, and concentration under physiological conditions. Furthermore, vapor pressure is considered to determine whether the formaldehyde releaser will be present as a vapor or aerosol upon inhalation. This concept is demonstrated using four examples that lead to different carcinogen classifications and MAK value derivations. Additionally, the releasers were evaluated for percutaneous absorption and sensitization.

**Supplementary Information:**

The online version contains supplementary material available at https://doi.org/10.1007/s00204-026-04489-7.

## Introduction

Formaldehyde is highly reactive, causing irritation and acute and chronic toxicity in target tissues (the eyes, respiratory tract, and skin) following direct contact. Moreover, formaldehyde is a local carcinogen after inhalation exposure. It induces nasal squamous cell carcinoma in laboratory animals and, albeit less convincing, nasopharyngeal carcinoma in humans. There is also limited evidence of an increased incidence of leukaemia in humans, but this is not supported by mechanistic or experimental findings. Based on sufficient evidence in humans and experimental animals, the International Agency for Research on Cancer (IARC) has classified formaldehyde as a human carcinogen (Category 1) (IARC 2006). According to the Classification, Labelling and Packaging (CLP) regulation, the European Chemicals Agency (ECHA) classified formaldehyde as a Category 1B carcinogen (carcinogen upon inhalation) based on sufficient evidence in experimental animals and limited evidence in humans (RAC 2012). After considering the mode of action, the German MAK Commission assigned formaldehyde to Carcinogen Category 4 (see the Supplementary information for the definition). Substances in this category are not expected to contribute to human cancer risk if exposure does not exceed the maximum workplace concentration (MAK value), and a non-genotoxic mode of action is of prime importance for carcinogenicity. Despite the induction of DNA lesions also at low doses, formaldehyde was classified as a Carcinogen Category 4 substance based on the assumption that an increase in mutagenicity and carcinogenicity can be prevented by ensuring that irritation and increased cell proliferation in target tissues do not occur. Based on this assumption, a MAK value of 0.3 ml/m^3^ (0.37 mg/m^3^) was established in 2000 (Greim 2002). Due to formaldehyde’s high reactivity, additional mechanistic evidence supports the conclusion that, upon inhalation, formaldehyde does not reach distant sites from the nose and is not expected to cause cancer or any other effects in tissues other than at the immediate portal of entry (Andersen et al. 2019; Hartwig et al. 2020).

Formaldehyde releasers are substances that release formaldehyde when they decompose in aqueous media. They are used, or were used in the past, as biocides in water-miscible metalworking fluids, adhesives, paints, disinfectants, and cosmetics. Qualitatively, formaldehyde releasers are assumed to have the same effects as formaldehyde itself.

According to ECHA, some formaldehyde releasers are evaluated as single substances, but not as a group. Since formaldehyde is classified as Category 1B Carcinogen, mixtures containing formaldehyde at a concentration of ≥ 0.1% w/w must be classified in the same category, following CLP guidance (ECHA 2024). However, the carcinogenicity of formaldehyde releasers upon inhalation depends on the rate at which formaldehyde is released in the respiratory tract. A more refined approach was chosen by the MAK Commission regarding both MAK value derivation and carcinogen classification, if possible by analogy to formaldehyde. As detailed in the four examples below, the quantitative risk was assessed by evaluating substance-specific inhalation data, if available, as well as data on the hydrolysis rate and formaldehyde release rate as a function of pH, temperature, and concentration. Furthermore, vapor pressure was considered to assess potential aerosol impaction. Similarly, notations for relevant percutaneous absorption and sensitizing potential were considered for all compounds (see Section III, Carcinogenic Substances and Groups of Substances Requiring Special Consideration, Formaldehyde Releasers, of the MAK and BAT Values List; DFG 2025).

## Illustration of the scientific approach using four examples

### Example 1: Tetramethylol acetylenediurea (TMAD)

**Fig. 1:**
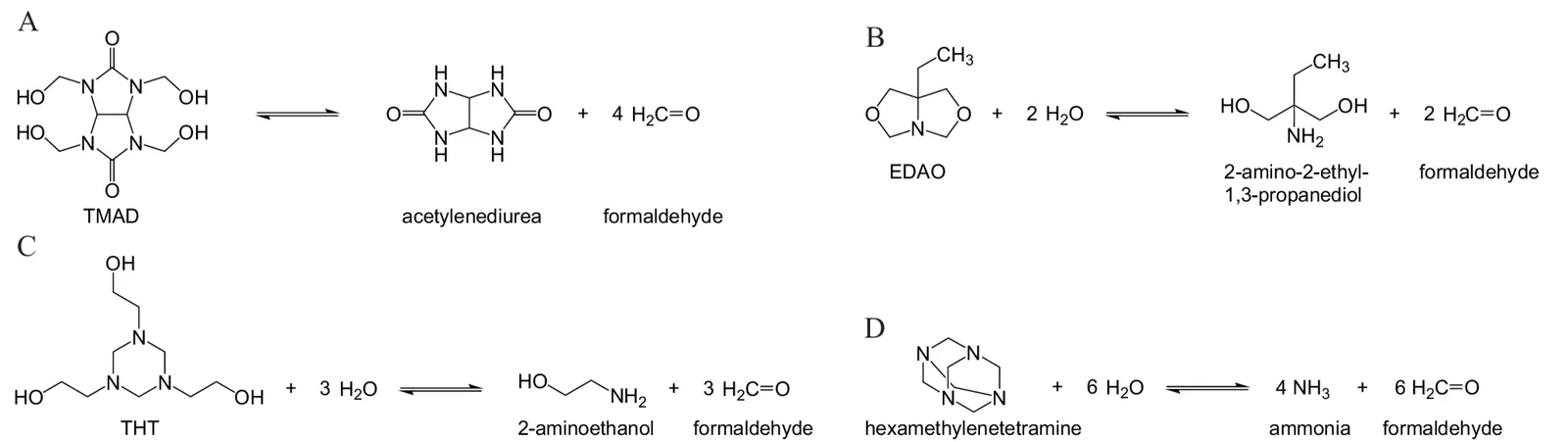
Formaldehyde release by tetramethylol acetylenediurea (TMAD) (**A**), 5-ethyl-3,7-dioxa-1-azabicyclo[3.3.0]octane (EDAO) (**B**), N, N′,N′′-tris(β-hydroxyethyl)hexahydro-1,3,5-triazine (THT) (**C**), hexamethylenetetramine (**D**)

In the case of TMAD, a MAK value was derived using substance-specific data, and, analogously to formaldehyde, TMAD was classified as a Carcinogen Category 4. Figure 1A illustrates the process by which formaldehyde is released from TMAD. TMAD has a very low vapor pressure and is expected to exist as an aerosol in the air. In a 28-day inhalation study with rats, slight laryngeal irritation was observed at a concentration of 21.7 mg/m^3^, the lowest concentration tested. This was probably due to the impaction of the aerosol used in the study. Irritation of the nasal cavity was observed at concentrations of 96 mg/m^3^ and higher. Acetylenediurea, the second hydrolysis product of TMAD, cannot be responsible for the effects induced by TMAD, as it did not cause eye or skin irritation and was nontoxic after oral administration of up to 1000 mg/kg body weight and day for 28 days. Therefore, the substance-specific data from the 28 day inhalation rat study with TMAD showing adverse effects on the upper respiratory tract were used for the MAK value derivation. This was based on standard assessment factors, including extrapolation from the LOAEC to the NAEC (factor 3), subacute exposure to chronic exposure (factor 2), and increased respiratory volume in the workplace (see Section I of the MAK and BAT Values List, DFG 2025). Furthermore, since the rat study revealed adverse effects on the larynx, an additional factor of three was applied to account for extrapolating from morphological observations in experimental animals to sensory irritation in humans, as detailed in Brüning et al. (2014). Using the preferred value approach, a MAK value of 0.5 mg TMAD/m^3^ for the inhalable fraction was derived (Hartwig and MAK Commission 2023a).

### Example 2: 5-Ethyl-3,7-dioxa-1-azabicyclo[3.3.0]octane (EDAO)

EDAO is one example where a MAK value was derived in analogy to formaldehyde in the absence of substance-specific inhalation data as the substance is present as a vapour, like formaldehyde. Based on the worst-case assumption that EDAO would undergo complete hydrolysis, forming 2-amino-2-ethyl-1,3-propanediol and two molecules of formaldehyde (Fig. 1B), a MAK value of 0.15 ml/m^3^ was derived for EDAO in analogy to the MAK value of formaldehyde of 0.3 ml/m^3^. Like EDAO, its hydrolysis product 2-amino-2-ethyl-1,3-propanediol causes irritation of the skin and eyes, but the effects are not as strong as those induced by the parent compound. Therefore, even assuming the complete hydrolysis of an EDAO concentration of 0.15 ml/m^3^, 2-amino-2-ethyl-1,3-propanediol would not cause any additional irritation. Additionally, EDAO has been classified as Carcinogen Category 4, in analogy to formaldehyde (Hartwig and MAK Commission 2025).

### Example 3: N, N′,N′′-Tris(β-hydroxyethyl)hexahydro-1,3,5-triazine (THT)

THT (hydrolysis, see Fig. 1C) is one example of a formaldehyde releaser for which a MAK value and a carcinogen classification in Category 4 based on formaldehyde release would not appear to be protective enough. In a 28-day inhalation study in rats, multifocal squamous metaplasia of the larynx and nose as well as degeneration of the bronchial epithelium were observed at the lowest concentration tested of 3 mg/m^3^ and above. Due to the severity of these effects, the study cannot be used to derive a NAEC or a MAK value. Moreover, a MAK value cannot be established in analogy to the MAK value of gaseous formaldehyde because the effects are exacerbated by aerosol impaction. This is supported by the low vapour saturation of THT of 0.005 ml/m^3^. Therefore, it is assumed that THT induces more severe local effects than formaldehyde or 2-aminoethanol because of aerosol impaction. The substance has thus been assigned to Carcinogen Category 2 (see supplementary information) with the footnote “Prerequisite for Category 4 in principle fulfilled, but insufficient data available for the establishment of a MAK or BAT value” (Hartwig and MAK Commission 2023b).

### Example 4: Hexamethylenetetramine

As shown in Fig. 1D, hexamethylenetetramine releases formaldehyde only under acidic conditions (Table 1). Based on the findings of a hydrolysis study that did not detect hydrolysis at a pH of 7.4, hexamethylenetetramine is expected to undergo very limited hydrolysis in the nasal mucosa (mucous pH 6.5). Even under slightly acidic conditions at pH 5.8, the substance’s half-life was 13.8 h. Based on these data, it is not assumed that the detoxification capacity of nasal tissue is exceeded. Thus, hexamethylenetetramine has not been classified as carcinogenic. Consistent with its low hydrolytic activity under physiological conditions, available data show only very slight skin irritation and no eye irritation. The standard approach was therefore applied to derive a MAK value by converting the NOAEL of 50 mg/kg body weight and day derived from oral studies into a workplace air concentration. The respective assessment factors include a species-specific correction for toxicokinetic differences between rats and humans (factor 4), extrapolation of data from animal studies to humans (factor 2), and the preferred value approach. This results in an MAK value of 20 mg/m^3^ (see Section I of the MAK and BAT Values List, DFG 2025, and Hartwig and MAK Commission 2026).

**Table 1:** Half-life of hexamethylenetetramine determined at different pH and 37.5 °C (Hartwig and MAK Commission 2026)

pH	2.0	4.6	5.8	7.4
Hydrolysis rate per hour	0.43	0.19	0.05	Not measurable
Half-life (h)	1.6	3.73	13.8	–

## Percutaneous absorption and sensitization

Additionally, the potential systemic availability of formaldehyde after percutaneous absorption of the parent compound is considered. First, the amount of parent compound transferred by dermal absorption into the blood is estimated. Then, assuming a complete release of formaldehyde into the blood as a worst case, the contribution to the endogenous level of free and bound (total) formaldehyde in blood is evaluated. This worst-case assumption can be modified by taking into account the stability of the formaldehyde releasing compound at the pH of blood. In case of a significant increase in total blood formaldehyde levels, the parent compound is designated with an “H” (skin notation).

While formaldehyde itself has been categorized as a contact allergen (Greim 2002), formaldehyde releasers are not generally categorized as contact allergens by the Commission but evaluated on a case-by-case-basis. To evaluate the sensitizing potential, the rate of hydrolysis under relevant conditions (e.g. pH) is taken into account to ascertain whether the resulting formaldehyde concentration can induce or elicit an allergic reaction. The pH dependence of the hydrolysis must be determined for each specific substance, as it cannot be derived in general. Regardless of the sensitizing effects caused by the release of formaldehyde (de Groot et al. 2009), it is always necessary to evaluate whether the substance itself shows a sensitizing potential.

## Conclusions

Formaldehyde releasers present an important source of exposure to formaldehyde if it is released under physiologically relevant exposure conditions. However, due to their diverse chemical properties, a standard approach for quantitative risk assessment cannot be applied to all substances, and they must be evaluated on a case-by-case basis. If substance-specific data are available, they are considered (Example 1). Otherwise, the extent of hydrolysis under physiological conditions is estimated, assuming complete hydrolysis when data are missing (Example 2). In this case, the MAK value is based on the amount of formaldehyde released, and the substance is classified as a Carcinogen Group 4. However, an MAK value may not provide adequate protection in cases of low vapor pressure with presumed aerosol impaction (Example 3). In such cases, a Carcinogen Group 2 classification is required. Conversely, if formaldehyde is not expected to be released, no carcinogen classification is warranted (Example 4). A case-by-case evaluation is also required for labeling with regard to percutaneous absorption and sensitization.

## Supplementary Information

Below is the link to the electronic supplementary material.

Supplementary Material 1

## Data Availability

No datasets were generated or analysed during the current study.
