# Supplementary material for: Scientific approach to derive occupational exposure limits for formaldehyde releasers
Source: Arch Toxicol. 2026 Jul 3;100(11):5323–7. doi: 10.1007/s00204-026-04489-7 (PMC13694579; doi:10.1007/s00204-026-04489-7)
Supplement: Supplementary file 1 [file 204_2026_4489_MOESM1_ESM.docx]

**Supplementary Material**

**Article Title: Guest Editorial: Scientific approach to derive occupational exposure limits for formaldehyde releasers**

**Journal Name:** Archives of Toxicology

**Author Names**: Sandra Michaelsen, Britta Laube, Rüdiger Bartsch, Stephan Baumgärtel, Dietmar Breuer, Rolf Grebenstein, Edgar Leibold, Ruth Lohmann, Hans Merk, Carsten Schleh, Manigé Fartasch, Michael Bader, Hans Drexler, Brunhilde Blömeke, Andrea Hartwig

**Corresponding author**: Andrea Hartwig (andrea.hartwig@kit.edu)

# Supplementary Information on the categories for carcinogenic substances (DFG 2025)

# Category 1

Substances that cause cancer in man and can be assumed to contribute to cancer risk. Epidemiological studies provide adequate evidence of a positive correlation between the exposure of humans and the occurrence of cancer. Limited epidemiological data can be substantiated by evidence that the substance causes cancer by a mode of action that is relevant to man.

# Category 2

Substances that are considered to be carcinogenic for man because sufficient data from long‐term animal studies or evidence from animal studies substantiated by evidence from epidemiological studies indicate that they can contribute to cancer risk. Limited data from animal studies can be supported by evidence that the substance causes cancer by a mode of action that is relevant to man and by results of in vitro tests and short‐term animal studies.

# Category 3

Substances that cause concern that they could be carcinogenic for man but cannot be assessed conclu­sively because of lack of data. The classification in Category 3 is provisional. Substances for which the available studies have yielded evidence of carcinogenic effects that is not sufficient for classification of the substance in one of the other categories. Further studies are required before a final decision can be made. A MAK or BAT value can be established, provided no genotoxic effects have been detected for the substance or its metabolites or the genotoxic effect is not the main effect.

# Category 4

Substances that cause cancer in humans or animals or that are considered to be carcinogenic for humans and for which a MAK value can be derived. A non-genotoxic mode of action is of prime importance and genotoxic effects play no or at most a minor part, provided the MAK and BAT values are observed. Under these conditions no contribution to human cancer risk is expected. The classification is supported espe­cially by evidence that, for example, increases in cellular proliferation, inhibition of apoptosis or distur­bances in cellular differentiation are important in the mode of action. The classification and the MAK and BAT values take into consideration the manifold mechanisms contributing to carcinogenesis and their characteristic dose‐time‐response relationships.

# Category 5

Substances that cause cancer in humans or animals or that are considered to be carcinogenic for humans and for which a MAK value can be derived. A genotoxic mode of action is of prime importance but is considered to contribute only very slightly to human cancer risk, provided the MAK and BAT values are observed. The classification and the MAK and BAT values are supported by information on the mode of action, dose-dependence and toxicokinetic data.

# References

DFG (Deutsche Forschungsgemeinschaft) (ed) (2025) List of MAK and BAT Values 2025. Maximum Concentrations at the Workplace and Assessment Values in Biological Material. Permanent Senate Commission for the Investigation of Health Hazards of Chemical Compounds in the Work Area, report 61. German Medical Science, Düsseldorf, https://doi.org/10.34865/mbwl_2025_eng
